# Successful diagnosis and treatment of ingested wooden toothpicks: Two case reports

**DOI:** 10.1097/MD.0000000000009710

**Published:** 2018-02-02

**Authors:** Nan Lin, Li Lin, Weihang Wu, Weijin Yang, Zhicong Cai, Jie Hong, Yu Wang

**Affiliations:** a Department of General Surgery, Dongfang Hospital, Xiamen University; b Department of General Surgery, Fuzhou General Hospital; c Clinical Institute of Fuzhou General Hospital, Fujian Medical University, Fuzhou, Fujian, China.

**Keywords:** diagnosis, treatment, wooden toothpick

## Abstract

**Rationale:**

Foreign-body ingestion is a common phenomenon and foreign bodies are mostly excreted in stool. Once sharp bodies are ingested without being realized, perforation of intestine is possible and misdiagnosis may be made. We report 2 toothpick ingestion cases that were both diagnosed accurately.

**Patient concerns:**

We present 2 cases of middle-aged persons who suffered from abdominal pain. They did not realize and provide any information of having the history of swallowing foreign bodies.

**Diagnoses:**

No serious problem was discovered in the examination and blood test. There were somewhere abnormal in computed tomography (CT) images and ultrasound (US). Then a toothpick was found penetrating the wall of intestine into the adjacent viscera in the laparotomy.

**Interventions:**

Both patients in the 2 cases were undergone operation to remove the toothpicks.

**Outcomes:**

The 2 cases’ prognoses were good.

**Lessons:**

When accepting patients with abdominal pain, suitable examining means and careful observation should be given to find easily ignored lesions. CT is recommended in the diagnostic process of swallowed foreign mass. When there is a vague place, US can be used for further diagnose.

## 1 Introduction

Foreign-body ingestion is a common phenomenon.^[1–6]^ Once sharp bodies, such as toothpicks, pins, and fishbone, are ingested, perforation of the intestine is possible and should be taken seriously. Toothpicks are ingested accidentally for various reasons—the ingestion happens to people who are edentulous, alcoholics, or who are used to picking their teeth.^[2,5,7–9]^ About 54% of patients cannot explain when or how the toothpicks get into their bodies.^[5]^ The symptoms of perforation are significantly different from relapsing blunt abdominal pain to typical peritonitis,^[1,4,5,7]^ mainly depending on the location where a toothpick lodges. Overall, the low morbidity and confusing clinical presentations tend to misguide physicians. A rapid and accurate diagnosis is necessary to avoid severe outcomes. Therefore, we present 2 cases of intestinal perforation caused by toothpicks.

## 2 Case report

Written informed consent was obtained from both patients for the publication of this manuscript and accompanying images.

### 2.1 Case 1

A 33-year-old man presented to our facility with chronic mild transferable abdominal pain 2 months after having a midnight snack. His vital signs were normal. Abdominal x-ray showed nothing abnormal before admission. The pain from epigastrium to hypogastrium could be relieved by the management of omeprazole pills (20 mg/day) for a few days. The mimic blunt pain relapsed after drinking. After admission, there was no obvious tenderness or mass in his abdomen during the body check-up. However, ultrasound (US) and abdominal computed tomography (CT) scans showed a linear shadow in the left liver lobe. The patient was taken to the operating room and underwent exploratory laparotomy, which revealed a wooden toothpick perforating intestinal wall with one end into the left liver lobe and the other end into the duodenum with surrounding inflammation and adhesion. The toothpick was successfully removed by snare extraction without complications. The patient made an uneventful postoperative recovery. When informed of this unusual finding during his follow-up, he recalled the probable accidental ingestion of a toothpick after drinking.

### 2.2 Case 2

**Figure 1:**
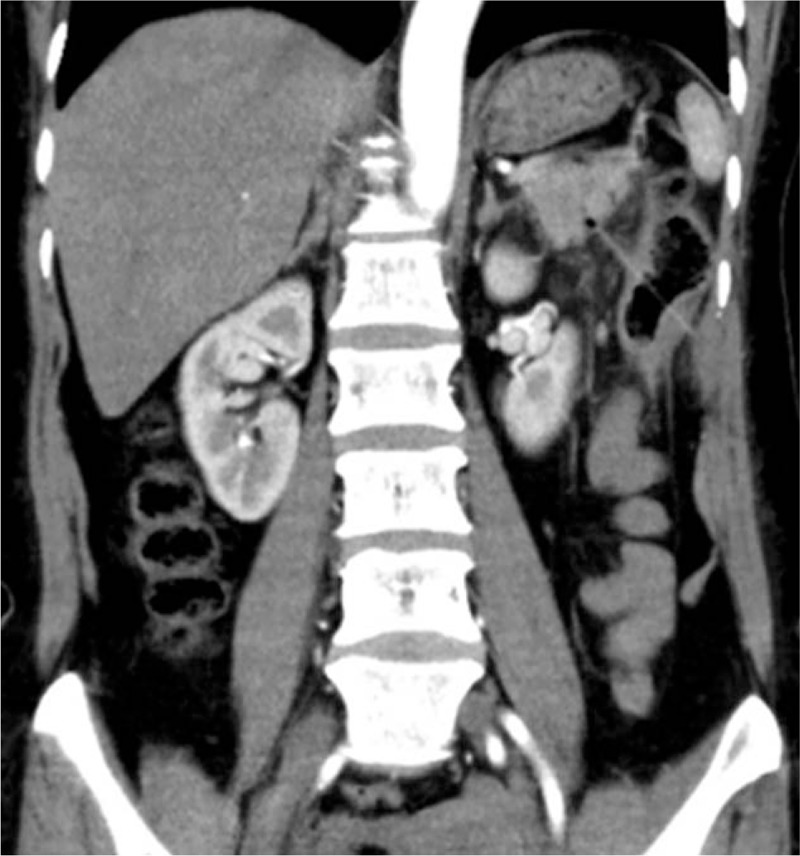
Coronal CT images show the continuous high-density point which can preliminarily tell us that a foreign body penetrates the wall of the intestine. CT = computed tomography.

**Figure 2:**
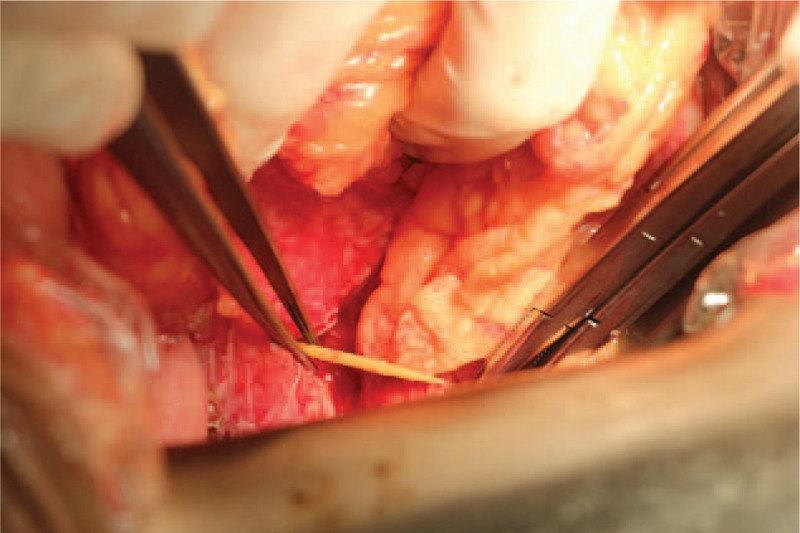
A bamboo toothpick is visible protruding from a perforated colon with minimal local reaction in the surgical screen.

**Figure 3:**
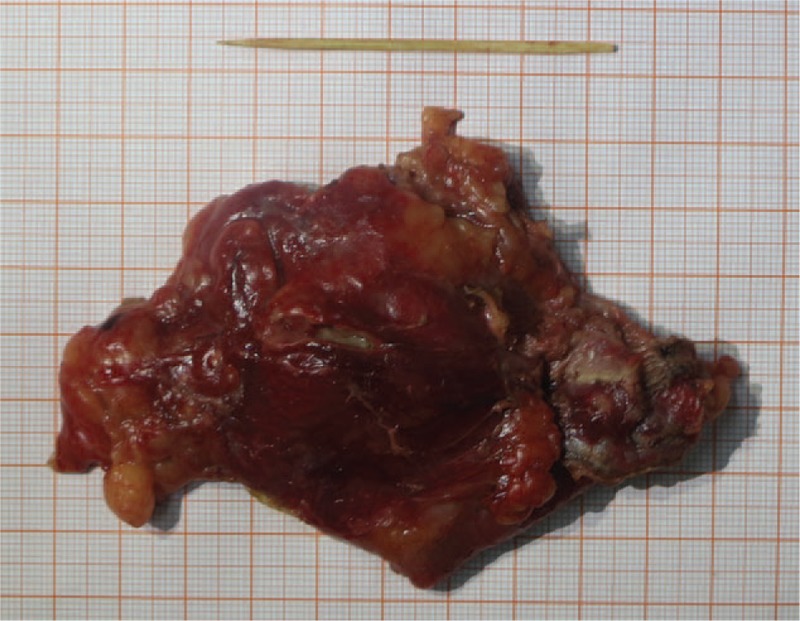
The toothpick is approximately 6 cm, which is the culprit of colon perforation.

A middle-aged woman suddenly suffered from a durative colic in left upper abdomen for 4 days without any radiation. There was deep tenderness in her left epigastrium without rebound pain and signs of peritonitis. Her vital signs were normal. No problem was found in the local hospital and then she was transferred to our hospital. Emergency laboratory evaluation revealed a blood amylase value of 105.0 U/L, a white blood cell count of 16 × 10^9^/L, and a granulocyte count of 12.1 × 10^9^/L (79.1%), while other relevant laboratory data were within reference values. The contrast-enhanced CT scan of the abdomen revealed a linear high-density shadow penetrating the descending colon and pointing to the tail of the pancreas with secondary surrounding inflammation (Fig. 1). The patient underwent emergency surgery. In the intraoperative probe, we found a bamboo toothpick penetrating the splenic flexure of the transverse colon wall into the pancreatic tail and obvious adhesion of the surrounding tissue (Fig. 2). The toothpick is approximately 6 cm (Fig. 3). Postoperative recovery was uneventful.

## 3 Discussion

The man in case 1 was misdiagnosed due to mild symptoms and careless imaging. Fortunately, the toothpick did not cause greater damage, and the patient had a good postoperative recovery. The woman in case 2 was not checked out any key problem in the first 4 days and was treated reasonably until coming to our department. However, any delay in diagnosis could have been potentially fatal. Both 2 patients in our report could not recall having ingested foreign bodies. After being inquired again after surgery, the male patient said that he had the habit of using a toothpick. Most of patients could not recall having swallowed the toothpicks.

Less than 1% of cases after swallowing a foreign body lead to perforation.^[5,9]^ The complaints vary according to the injured parts and adjacent organs. The locations of toothpicks before removal were the esophagus (2%), stomach (20%), duodenum (23%), small intestine (18%), and large intestine (37%).^[2,5]^ The main impressionable adjacent viscera include liver, pancreas, kidney, and vasculature.^[4,5,10]^ Complications of perforation include fistula, sepsis, bleeding, and even death.^[1,11]^ The location that a toothpick lodges determines the symptom.

Intensive patients were directly referred for exploratory surgery, and then the foreign bodies were found. The remaining cases were examined out the existence of foreign bodies with more conservative methods, such as x-ray, US, magnetic resonance imaging (MRI), CT, and endoscopy.^[5,9,12]^ However, the sensitivity of these methods depends on the organs examined. X-ray is easily examined and can reveal indirectly symptoms of digestive tract injury (e.g., cavity-free gas in the thorax and abdomen).^[13,14]^ As for the abdominal x-ray, it can be used for initial screening and the diagnostic significance is small. The sensitivity of US in the diagnosis of damage to the solid viscera is high, and US has the advantage of real-time dynamic observation from different angles.^[14,15]^ CT and MRI have superior sensitivity in any gastrointestinal injury. Through observing the continuing fixed plane angle, it is easy to find the differential part if the imaging readers report the result carefully and prudently.^[15]^ But MRI is not only more expensive but also less accurate than CT. On the other hand, longer time should be used in MRI. Endoscopy can directly show the location of toothpicks in the early stage, and corresponding treatment can be made at the same time.^[3,5]^

In conclusion, when accepting patients with abdominal pain, suitable examining means and careful observation should be given to find easily ignored lesions. CT is recommended in the diagnostic process of swallowed foreign mass. When there is a vague place, US can be used for further diagnose. If necessary, we can choose endoscopic to diagnose and treat at the same time. In case of emergency, laparotomy is necessary. A more accurate diagnosis and corresponding treatment should be made to reduce the pain of the patient and help them recover without delay.
